# Metoclopramide-induced hyperprolactinemia during pregnancy and lactating alters chondrocyte proliferation and apoptosis in the murine femoral and tibial epiphyseal growth plate

**DOI:** 10.1016/j.clinsp.2026.101016

**Published:** 2026-06-10

**Authors:** Osvaldo P. Araujo-Jr, Regina C.T. Gomes, Ariadne S.L. Araujo, Edmund C. Baracat, Ricardo S. Simões, Manuel de J. Simões, José M. Soares Junior

**Affiliations:** a Obstetrics and Gynecology Department, Universidade Federal de São Paulo (UNIFESP), São Paulo, SP, Brazil; b Obstetrics and Gynecology Department, Faculdade de Medicina da Universidade de São Paulo (FMUSP), São Paulo, SP, Brazil; c Morphology and Genetics Department, Universidade Federal de São Paulo (UNIFESP), São Paulo, SP, Brazil

**Keywords:** Hyperprolactinemia, Epiphyseal growth plate, Cell apoptosis and proliferation, Lactating, Mice

## Abstract

**Study design:**

Experimental study about hyperprolactinemia on the Epiphyseal Growth Plate (EGP).

**Objectives:**

Evaluated the EGP of the lactating female with hyperprolactinemia induced by metoclopramide and of the physiological lactating female.

**Methods:**

Ten (10) females received 0.2 mL of saline solution at 0.9% (control group, CTR), and ten (10) females received 200 µg/day of metoclopramide (experimental, HPRL). Treatments lasted for 79 consecutive days (50 days of induction of HPRL, 19 days of gestation, and 10 days of breastfeeding). On the 79th day, the lactating female mice were euthanized and the knees were removed and fixed in 10% formaldehyde for 24 hours and decalcified bone for immuno-histochemistry (TUNEL and Ki67) analysis, and use Masson's trichrome. Data were statistically analyzed by the Mann-Whitney test (p < 0.05).

**Results:**

Apoptosis of chondrocytes was higher in the EGP (femur and tibiae) of the lactating female-HPRL compared to the EGP (femur and tibiae) of the lactating female-CTR (p < 0.05). Cell proliferation of chondrocytes was significantly higher in the EGP in the femur compared to the EGP in the femur of lactating females-CTR (p < 0.05), however, the EGP in the tibiae (HPRL vs. CTR) was not significant, p > 0.05.

**Conclusions:**

The present study revealed excess cell death (TUNEL) of chondrocytes, consisting of 2-fold in the EGP of the femur and 4-fold in the EGP of the tibiae of a lactating female with hyperprolactinemia induced by metoclopramide, with greater involvement of the tibiae in relation to the femur, which is a fact that can lead to an imbalance between chondrogenesis and osteogenesis.

## Highlights

• Metoclopramide-induced hyperprolactinemia exacerbates chondrocyte apoptosis in the growth plates of lactating mice.
• Tibial growth plates show a 4-fold increase in apoptosis, indicating a greater vulnerability than femoral plates.
• Chondrocyte death occurred prematurely in the proliferative zone, disrupting growth plate architecture.
• The imbalance between cell death and proliferation suggests a mechanism for HPRL-linked bone loss.

## Introduction

The prevalence of Hyperprolactinemia (HPRL) is 9%–17% in reproductive-age women.^1^^,^^2^ Hyperprolactinemia can occur physiologically due to pregnancy and lactating or pathological or medication causes.^2^

Prolactin (PRL) is synthesized and secreted by lactotrophs of the adenohypophysis.^3^ In the past, it was known to control milk secretion after birth, but its systemic action is now recognized, including its effect into epiphyseal growth plate (EGP) and bone in humans and murine.^4^^,^^5^

In animal models for Hyperprolactinemia induced by metoclopramide (HPRL) acting negatively on ovarian function, leading to decreased secretion of ovarian steroids (progesterone and estradiol)^2^^,^^5^^,^^6^ a fact that can lead to a condition similar to hypogonadism. However, there are several unanswered questions about the influence of HPRL on the EGP of pregnancy and lactating.

It is known that the EGP and bone are highly specialized connective tissues, and although both have many varied functions, some of these functions are similar and correlated, as long bones such as the tibiae and femur undergo endochondral ossification. Endochondral bone growth is the main mechanism that determines skeletal structure, bone morphology and bone mineral accretion. This type of ossification is a complex process, as it involves genetic and hormonal factors responsible for the proliferation, differentiation, maturation and apoptosis of chondrocytes in the EGP, a fact that histologically results in the formation of three zones (metaphysis to diaphysis: resting, proliferative and hypertrophic).^7^ In this context, prolactin can directly affect the cartilage of the EGP, influencing chondrocyte proliferation, differentiation, and function, and impacting bone growth.^7^ Furthermore, it should be noted that cartilage is avascular, being nourished by the surrounding connective tissue (perichondrium) and does not present nerve and lymphatic vessels; thus, damage to the cartilage is fatal and consequently to the bone tissue.^8^ Furthermore, throughout an individual's life, the EGP normally decreases in thickness until only the disc remains in humans. This process is called fusion or closure of the EGP. The fusion of the EGP is related to sexual maturation in humans and murine.^8^, ^9^, ^10^ However, in humans, this fusion occurs around 20 years of age, and determines the growth arrest of the individual in adulthood, varying between individuals and bones. In murine (rats and mice), sexual maturity occurs at 2 months of age, and the fusion of the EGP occurs later. A recent study demonstrated that although the mouse EGP calcifies with age, it remains calcified cartilage for an extended period without being replaced.^10^ For this reason, mice and rats are frequently used as animal models to study the EGP and bone development, due to their genetic similarity to humans, and a relatively short generation time, allowing for faster breeding, which allows the evaluation of therapeutic interventions.^11^

Pregnancy and lactation are complex physiological processes that affect female physiology by presenting different hormonal profiles to meet the additional nutritional demands of fetuses and neonates.^9^^,^^12^^,^^13^ Research reports decreased Bone Mineral Density (BMD) in physiological HPRL during pregnancy and lactation in women, sheep and rodents. BMD can lead to osteoporosis.^14^ Osteoporosis is a common skeletal disorder characterized by reduced bone strength due to decreased BMD and bone quality.^13^, ^14^, ^15^, ^16^

During pregnancy and lactation, the role of PRL is to accelerate maternal bone reabsorption to make nutrients and calcium available to the fetus for the formation of the fetal skeleton and the neonate, respectively.^14^, ^15^, ^16^

It is known that normally, the thickness of maternal EGP is the result of a balance between chondrogenesis and osteogenesis, that is, between the inhibition and induction of chondrocyte apoptosis.^17^ Effects of metoclopramide-induced HPRL on EGP during pregnancy/lactating are not known. For this reason, the authors carried out the present study.

The main goal of this study was to analyze the apoptosis marker (TUNEL) and cell proliferation marker (Ki-67) the chondrocytes on the epiphyseal growth plate of the femur and tibiae of the lactating female with metoclopramide-induced HPRL and of the lactating female physiological.

## Methods

Twenty virgin female mice (albino Swiss) and five fertile male mice (Swiss albino), both (3-month-old, *Mus musculus*, and weighing 35‒40 g), were given food and water ad *libitum* at room temperature (22 °C) under artificial light with a 12-hour light: dark photoperiod cycle. The experiments were performed at the Histology and Structural Biology Discipline of the Federal University of São Paulo (UNIFESP, Brazil). The sample size was determined based on previous studies.^5^

### Ethics

This study was approved by the Animal Experimentation Ethics Committee and the scientific board of the Federal University of São Paulo (UNIFESP, Brazil), Report n° 6082130319. The authors comply with the ARRIVE guidelines as well as with other guidelines established by the Canadian Council on Animal Care (CCAC), the Brazilian School of Animal Experimentation (Colégio Brasileiro de Experimentação Animal ‒ COBEA), and the National Council for Control of Animal Experimentation (Conselho Nacional de Controle de Experimentação Animal ‒ CONCEA).

#### Experiment 1 (induce the hyperprolactinemia)

The main goal of this study was to analyze the epiphyseal growth plate (femur and tibiae) under the influence of the hyperprolactinemia, to achieve the objective in this first phase of the experiment was performed the treatments for the induction the hyperprolactinemia.

#### Treatment

Then, 20 female mice were randomly divided into two groups with 10 female mice each: control group (CTR): ten (10) female mice received subcutaneous injections of 0.2 mL of saline solution (vehicle) for 50 consecutive days and experimental group (HPRL): ten (10) female mice received subcutaneous injections of 200 µg/day metoclopramide dissolved in 0.2 mL of saline solution for 50 consecutive days. The female mice were always treated at 12:00 noon.

#### Experiment 2 (pregnant)

On the 50th day, colpocytological examinations of the vaginal smears were conducted 1 hour after the last injection. Then, the female mice in the control group (estrus phase) and the female mice in the experimental group (estrus phase) were placed for mating, in the ratio of three females for each fertile male. Mating was proven by the presence of a vaginal plug.^18^ In this way, the authors obtained the pregnant female group with physiological hyperprolactinemia (CTR) and the pregnant female group with metoclopramide-induced HPRL. The pregnant females continued to receive the treatments with saline solution (CTR group) or metoclopramide (HPRL group) during pregnancy, respectively.

#### Experiment 3 (lactating females)

After birth, each lactating female mouse (mother) remains with her offspring for lactation (the offspring receives only breast milk for 10 consecutive days during lactation). The offspring were weaned at 10 days (which were part of another study). In this way, the authors obtained the lactating female group with physiological hyperprolactinemia (CTR) and the lactating female group with metoclopramide-induced HPRL. The lactating females continued receiving the treatments with saline solution (CTR group) or metoclopramide (HPRL group) during lactation, respectively.

Total treatment with saline or metoclopramide was 79 days (50 days = treatments for the induction of hyperprolactinemia) + (19 days = gestation) + (10 days = breastfeeding).

Afterward, the lactating females were euthanized. The euthanasia was performed by deepening the subcutaneous tissue in association with a muscle relaxant plus anesthetic (xylazine 20 mg/kg plus ketamine 100 mg/kg).^18^ Finally, the knee (EGP of the femur and tibiae) of each female was removed and fixed for 24 hours in 10% formaldehyde in phosphate-buffered saline (PBS, pH 7.4) and decalcified for histological processing.^5^

### Histological processing

After fixation, the knees (EGP of the femur and tibiae) were immersed in 10% Ethylenediamine Tetra Acetic Acid (EDTA) in phosphate-buffered saline (PBS, pH 7.4) buffer to decalcify the bones; the solution was changed every two days until decalcification was complete at room temperature. Then, the knees were processed according to the standard technique for embedding in paraffin in histology equipment (Lupe Ltda, Brazil).

**Fig. 1:**
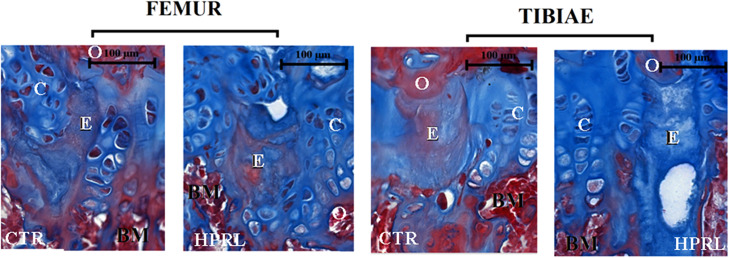
Longitudinal section of the epiphyseal growth plate (EGP) of the femur and tibiae from lactating female-HPRL group compared to the EGP from lactating female-CTR group. Note the EGP Masson's trichrome stain to differentiate collagen fibers, particularly in areas of erosion process (E). Note the chondrocytes (C) embedded in cartilaginous extracellular matrix (ECM) into EGP, the blue color represents type II collagen fibers in cartilaginous tissue, and the red color represents type I collagen fibers in bone tissue (O). Note absence of chondrocytes (C) or osteocytes (O) into areas of erosion (E). This suggests a change in the composition of the ECM in areas of erosion, but there is no bone tissue per se. The analyses were performed at 400 × magnification (Bar = 100 µm).

Each paraffin-embedded knee (EGP of the femur and tibiae) was cut into two sections/knee of 3 μm on a Minot microtome LEICA ‒ RM 2145 (Leica, USA), the sections were adhered in silanized slides at 5% for the Immunohistochemistry method (IHC), and the sections were adhered to histological slides. The histological slides were stained with Masson's trichrome, which is a histochemical staining technique that differentiates different types of collagens. The authors use it to differentiate bone tissue from cartilage tissue (Fig. 1).

### Immunohistochemical for the detection of Ki67 and TUNEL

Initially, the paraffin-embedded knee (sections were submitted to the standard protocol of deparaffinization (3 xylol baths for 5 mins each), rehydrated (3 ethanol baths for 5 mins each), and formic acid was added (one bath for 3 mins). The sections were washed several times in running water and then washed three times in distilled water. Afterward, the sections were submitted to the protocol of Immunohistochemical (IHC): IHC was performed using primary antibodies (rabbit anti-rat monoclonal) for detection: Ki-67 (Clone MiB-5) (dilution 1:50, catalog # M724801-8, DakoCytomation, Denmark) and Marker for cell death by DNA fragmentation ‒ TUNEL (TdT-mediated dUTP-biotin Nick End Labeling) method (in Situ Cell Death Detection Kit, catalog # 11684817910 Roche, USA).

### Detection of Ki-67 by immunohistochemistry

**Fig. 2:**
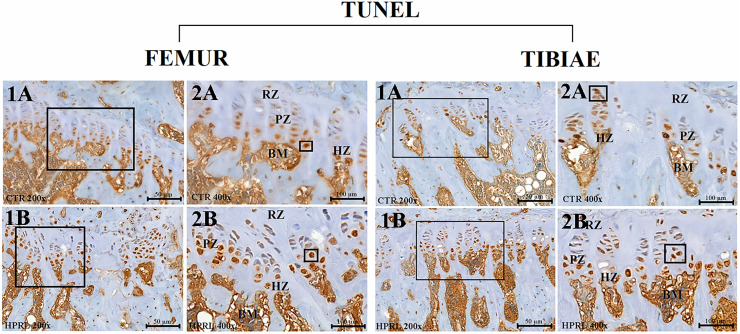
Representative photomicrographs of the immunohistochemistry for cell apoptosis marker (TUNEL). Longitudinal section of the epiphyseal growth plate (EGP) of the femur and tibiae from lactating female- HPRL group compared to the EGP from lactating female-CTR group. The EGP is divided into Reserve Zone (RZ), Proliferative Zone (PZ) and the Hypertrophic one (HZ). Note the intense brownish staining indicating the chondrocytes embedded in cartilaginous extracellular matrix into EGP (small rectangle) corresponds to immunopositive cells to TUNEL. Fig. 1A and 1B, the analyses were performed at 200 × magnification (Bar = 50 µm) and Fig. 2A and 2B correspond to the area within the small rectangle of Fig. 1A and 1B, respectively and the analyses were performed at 400 × magnification (Bar = 100 µm).

1) The antigenic recovery; 2) Endogenous peroxidase; 3) Non-specific sites were blocked; 4) Sections were incubated with primary antibody diluted in PBS; 5) Next, sections were incubated for 30 minutes with the post primary, and again incubated with the avidi/biotin complex for 30 mins (VECTASTAIN® Elite ABC kits anti-rabbit (catalog # PK-6101, Vector Laboratories, USA, 6) Diaminobenzidine chromogen (DAB) (DakoCytomation, Denmark), and 8) Counterstained with hematoxylin Harris for 2 minutes. Negative controls were obtained by replacing the primary antibody with the respective concentration of non-immune IgG from the same species. The entire protocol was published by Araujo et al., 2023[18] (Fig. 2).

### Detection of nuclear DNA degradation by the TUNEL assay

**Fig. 3:**
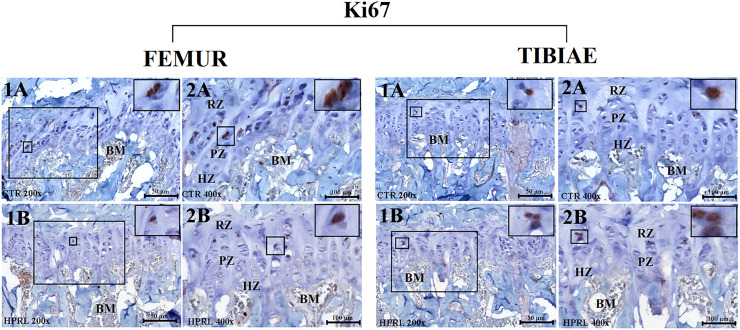
Representative photomicrographs of the immunohistochemistry for cell proliferation marker (Ki67). Longitudinal section of the epiphyseal growth plate (EGP) of the femur and tibiae from lactating female- HPRL group compared to the EGP from lactating female-CTR group. The EGP is divided into Reserve Zone (RZ), Proliferative Zone (PZ) and the Hypertrophic Zone (HZ). Note the intense brownish staining indicating the chondrocytes embedded in cartilaginous extracellular matrix into EGP (small rectangle) corresponds to immunopositive cells to Ki67. Fig. 1A and 1B, the analyses were performed at 200 × magnification (Bar = 50 µm) and Fig. 2A and 2B correspond to the area within the small rectangle of Fig. 1A and 1B, respectively and the analyses were performed at 400 × magnification (Bar = 100 µm).

The sections were stained using the in-situ marker for cell death by DNA fragmentation detection kit (TdT-mediated dUTP-biotin Nick End Labeling) method (in Situ Cell Death Detection Kit, catalog # 11684817910 Roche, USA) as recommended by the manufacturer. For negative controls, the TdT enzyme was substituted by distilled water. The entire protocol was published by Araujo et al., 2023[18] (Fig. 3).

### Semi-quantification and analysis methods

All sections were digitized using a computerized system; initially, the sections were scanned using a Histech 3D scanner (Budapest, Hungary) and viewed and then photographed using Panoramic Viewer software. Afterward, ten fields (at a magnification of 100 × to 400 ×) were selected in order to cover the entire length of the section, allowing a complete analysis of each knee (EGP of the femur and tibiae). Morphological and semi-quantification analyses were performed using the Image-Pro Plus7 software (software from Media Cybernetics, USA). For cell proliferation marker (Ki-67) and cellular death marker by DNA fragmentation (TUNEL), the results are expressed in % positive nuclei/total nuclei in μm^2^. The analyses of photomicrographs of the immunohistochemical sections were conducted by two histologists who were on a blind-study.

### Statistical analysis

The data were tested for normality using the Shapiro-Wilk test, and the results indicated that the data do not follow a normal distribution. For this reason, the unpaired Mann-Whitney test was used in the statistical analyses to compare the groups. The data are presented as the mean ± Standard Deviation (SD). All statistical tests were performed using GraphPad Prism software version 3.0 for Windows (GraphPad Software, San Diego, CA, USA). The significance level was set at p ≤ 0.05 for all statistical tests.

## Results

### Histological

The morphological analyses revealed disorganization in the arrangement of the chondrocytes within the three zones (rest zone, proliferation zone, and hypertrophic zone) that form the EGP from the lactating female-HPRL group were compared to the EGP from the lactating female-CTR group.

Furthermore, Masson's trichrome allows us to differentiate bone tissue from cartilaginous tissue by the type of collagen in the EGP (femur and tibiae). This fact allowed us to visualize deposits of type I collagen (the main component of the extracellular matrix of bone tissue) and type II collagen (the main component of the extracellular matrix of cartilage tissue). The authors observed an absence of chondrocytes or osteocytes in the area, which we call the area of an erosive process, and a mesenchymal of type I collagen and type II collagen, in the three zones that form the EGP, being more present in the EGP from the lactating female-HPRL group compared to the EGP from the lactating female-CTR group (Fig. 1).

### Semi-quantification and immunolocalization

The results of the semi-quantification of the TUNEL (marker for cell death, DNA fragmentation) and Ki67 (marker for cell proliferation) in EGP (femur and tibiae). The results are expressed in percentage (%) and summarized in Fig. 2, Fig. 3 and Table 1.

**Table 1:** Immunohistochemistry for the apoptosis marker (TUNEL) and cell proliferation marker (Ki-67) in epiphyseal growth plate of the femur and tibiae of lactating female with metoclopramide-induced hyperprolactinemia and lactating female with physiological hyperprolactinemia.

% positive nuclei/total nuclei in μm^2^						
Marker	Femur			Tibiae		
	CTR	HPRL	p-value	CTR	HPRL	p-value
Ki-67	0.66±0.36	2.45±0.95^a^	0.005	0.96±0.55	1.76±1.21	0.31
TUNEL	2.18±0.79	4.96±1.77^a^	0.0001	2.39±1.20	8.00±0.67^a^	0.0001
Ki67/TUNEL	0.33±0.20	0.52±0.31	0.41	0.55±0.49	0.22±0.15	0.15

Note: n = 10 female/group. Data are presented as means ± Standard Deviation (SD). Data were statistically analyzed by unpaired Mann-Whitney test (p < 0.05). The ^a^ indicate significant differences among groups: p < 0.05 = significant.

Lactating female-CTR group (CTR) and lactating female-HPRL group (HPRL).

Ki67 (cell proliferation marker): ^a^ p < 0.05 compared to HPRL vs. CTR in the Femur, and TUNEL (cell death marker, apoptosis): ^a^ p < 0.05 compared to HPRL vs. CTR in the femur and tibiae, and homeostasis index: the difference between the analyzed groups was not significant.

#### TUNEL (cell death marker, apoptosis)

- Fêmur: the increase of TUNEL immunopositive cells was significantly greater in the EGP from the femur of the lactating female-HPRL group compared to the EGP of the femur from the lactating female-CTR group, Fig. 2 and Table 1.
- Tíbiae: the increase of TUNEL immunopositive cells was significantly greater in the EGP of the tibiae from the lactating female-HPRL group compared to the EGP of the tibiae from the lactating female-CTR group, Fig. 2 and Table 1.

#### Ki67 (cell proliferation marker)

- Femur: number of Ki67 immunopositive cells was significantly higher in the EGP of the femur from the lactating female-HPRL group compared to the EGP of the femur from the lactating female-CTR group, Fig. 3 and Table 1.
- Tíbiae: number of Ki67 immunopositive cells was not significant in the EGP of the tibiae from the lactating female-HPRL group compared to the EGP of the tibiae from the lactating female-CTR group, Fig. 3 and Table 1.

### Homeostasis index

The homeostasis index represents the balance in the proliferation and apoptosis of a tissue. The Ki-67/TUNEL was not significant in the EGP (Femur and Tibiae) from the lactating female-HPRL group compared to the EGP from the lactating female-CTR group (Table 1).

## Discussion

The present study revealed excess chondrocyte death in the EGP (Femur and Tibiae) from the lactating female with metoclopramide-induced HPRL in relation to the EGP (Femur and Tibiae) from the lactating female with physiological HPRL. These data are evidence that constant exposures to prolactin (HPRL state) may contribute to the imbalance between bone production/resorption, which occurs at the EGP/bone tissue level, respectively.^19^

Non-lactation-related HPRL is a common cause of amenorrhea in women of childbearing age, and the result in the decrease in Gonadotropin-Releasing Hormone (GnRH) caused by high prolactin levels can result in decreased Bone Mineral Density (BMD). In patients with HPRL, the changes in BMD may be caused indirectly by inhibition of the GnRH-gonadal axis due to increased prolactin levels or through the direct action of prolactin on osteoblasts and possibly in the osteoclast cells.^1^^,^^12^, ^13^, ^14^, ^15^, ^16^

The PRL and its receptors are present in the chondrocytes and osteocytes.^5^ In cartilage of the EGP, the role of PRL is to stimulate the synthesis of components of the extracellular matrix, such as proteoglycans and type II collagen, by chondrocytes and mesenchymal cells derived from the bone marrow. This is a fact that inhibits the apoptosis of chondrocytes; moreover, the PRL is present in the synovial fluid where it is produced by synovial cells and can influence cartilage survival. In addition, it can act as a cytokine and exert immunoregulatory effects.^20^, ^21^, ^22^ In the bone, PRL participates in cell proliferation and bone mineralization. In addition, the presence of the Prolactin Receptor (PRL-R) in osteoblasts suggests that it has a role in bone formation and the maintenance of bone mineral density (BMD).^5^^,^^23^

During pregnancy, there is an increase in serum PRL levels (physiological HPRL) in the mother in response to increased placental steroids, with low transfer of PRL from mother to fetus and vice versa. Likewise, amniotic fluid contains a large amount of PRL, and finally, the role of PRL in lactation is unquestionable.^24^^,^^25^

In pregnancy and lactating, PRL accelerates bone resorption in the mother to provide adequate nutrients and calcium available to the fetus for the formation of its skeleton and contribute to longitudinal bone growth in the newborn, respectively.^16^^,^^25^, ^26^, ^27^, ^28^ It is observed that, prolactin also stimulates intestinal calcium absorption and trabecular bone resorption to provide adequate calcium for milk production during lactating, the latter culminates in low BMD in lactating mothers.^12^, ^13^, ^14^, ^15^, ^16^^,^^24^

Interestingly, research has shown that there is an increase in maternal endochondral bone growth during pregnancy and lactating in humans, sheep, and rodents. It is speculated that the process of maternal bone lengthening may help replenish maternal bone trabeculae to retain bone mass and/or compensate for the reduced BMD with maternal bone loss due to increased bone resorption during this period.^1^^,^^12^

In situations of hypoestrogenism/hypoganodism, such as HPRL (Hypothalamic-Pituitary-gonadal axis), biological mechanisms can affect the bone tissue level and, as a consequence, there is no complete blockade of the RANK/RANK-L system and cytokines.^29^^,^^30^ The RANK/RANK-L binding promotes the induction of osteoclastogenesis and the activity of osteoclasts, i.e., bone resorption.^29^ This fact can increase the rate of bone remodeling, prolonging the lifespan of osteoclasts and shortening the life of osteoblasts, leading to an imbalance between osteoclastogenesis (bone resorption) and osteoblastogenesis (bone formation).^29^, ^30^ Future molecular biology and electron microscopy studies will be carried out with the aim of clarifying the interference of HPRL in the RANK/RANK-L system.

The histological analysis results revealed changes in the architecture of the EGP (Femur and Tibiae), and it was possible to view areas without the presence of chondrocytes (areas in erosion) in the three zones that form the EGP (resting zone, proliferation zone and hypertrophic zone).

The areas in erosive process were greater in the EGP (Femur and Tibiae) from the lactating females-HPRL in relation to the EGP (Femur and Tibiae) from the lactating females-CTR. Masson's trichrome staining allowed the differentiation of collagen fibers in areas subjected to the erosion process. This suggests a change in the composition of the cartilaginous Extracellular Matrix (ECM) in these areas, where there was a mixture of type II collagen fibers (cartilage ECM) and type I collagen fibers, mainly components of the bone ECM, but without bone tissue per se. These results corroborate a recent study that demonstrated that it remains as calcified cartilage for a prolonged period without being replaced by bone, although mouse EGP calcifies with age in mice.^10^ The present results suggest that lactating may accelerate this process of calcification of the cartilaginous ECM; however, future studies with the largest number of animals are needed to confirm or refute this hypothesis.

The appearance of areas in the erosion process in the EGP from the lactating females-HPRL may partly be due to the limited capacity of the cartilage to regenerate, the mobility of chondrocytes, and the mature chondrocytes to proliferate. Therefore, it leads to the formation of new blood vessels in the local in altered or damaged cartilage in adults, which stimulates cartilage calcification. For this reason, these results led us to investigate cell death and cell proliferation of chondrocytes to determine the causes of erosions in the EGP of the lactating female mice from both groups.

The immunoexpression results showed excess chondrocyte death (TUNEL) in the three zones that form the EGP (Femur and Tibiae), starting early in the resting zone and extending through the proliferation zone and the hypertrophic zone in the lactating female group.

The increase in chondrocyte death was 2-fold in the EGP of the femur and 4-fold in the EGP of the tibiae from the lactating females-HPRL in relation to the EGP of the femur and EGP of the tibiae from the lactating females-CTR, respectively. A small chondrogenic activity (Ki67) was verified in the proliferative zone in the EGP (Femur and Tibiae) from the lactating female- HPRL in relation to the EGP (Femur and Tibiae) from the lactating females-CTR. However, this was derisory compared to the excess cell death, which is a fact that can lead to an imbalance between chondrogenesis and osteogenesis.

These results show serious impairment of the EGP (Femur and Tibiae) from the lactating females-HPRL. The excess cell death of chondrocytes in the EGP can interfere with the process of differentiation and proliferation of chondroblasts, which is a fact that can lead to degeneration of the EGP.

In addition to these classic endocrine effects, prolactin plays a role in regulating humoral and cellular immune responses, acting as an immunomodulator and also in angiogenesis.^31^, ^32^, ^33^, ^34^

The low cell density (hypocellularity), which is due to excess cell death, the chondrocytes found in the EGP from the lactating females-HPRL may result in loss of adhesion chondrocyte/chondrocyte, chondrocyte/extracellular matrix. The loss of the interaction between cells and the cell-extracellular matrix can interfere with the signaling for chondrocyte survival and lead to an increase in the content of IL-1 synthesized by chondrocytes, which can negatively regulate SOX-9, resulting in the inhibition of the synthesis of components of the extracellular matrix.^31^, ^32^, ^33^, ^34^ Consequently, this compromises the biomechanical functions of providing structural support and resistance to deformation leading to degeneration of cartilage and bone, which is a fact that can lead to an inflammatory process.

Furthermore, a complex network of signaling pathways regulates the development of epiphyseal chondrocytes (proliferation and differentiation), as well as genetic factors and various endocrine, paracrine and autocrine agents such as growth hormone, sex hormones (estrogen, progesterone and androgen). Hormones: Indian hedgehog protein duo (Ihh), Parathyroid Hormone-related Peptide (PTHrP), Parathyroid Hormone (PTH), prolactin, Growth Hormone (GH), IGF1 and 2, locally produced factors (FGF2, EGF, PDGF, TGFβ), beta-catenin, bone morphogenetic proteins, insulin-like growth factor, iodothyronine deiodinase, leptin, nitric oxide and vitamin D metabolites are also involved in the health of adult EGP and the maintenance of bone renewal.^35^, ^36^, ^37^, ^38^, ^39^

As mentioned, the risk of osteoporosis can occur in women after pregnancy/lactating by taking into account factors such as age, hormonal levels, nutritional status, and genetics. This osteoporosis can be temporary and reversible. Similarly, the present study’s results on Hyperprolactinemia (HPRL) after pregnancy/lactating prove that it can be reversible in female mice, since chondrocyte death occurs at the end of the hypertrophic zone of the EGP. This chondrocyte death is physiologically normal, which is a fact that did not occur in the EGP from female mice in the HPRL group induced by metoclopramide after pregnancy/lactating (altered sex hormone levels and constant prolactin levels). In this group, chondrocyte death occurred early in the proliferative zone and in the hypertrophic zone in most females, corroborating the morphological data that show disordered cellular organization in the EGP, which is a fact that can lead to plate degeneration and compromise bone formation and maintenance. There is still a need for further investigation into the complications caused by excess chondrocyte death of the EGP (femur and tibiae) in pregnant/lactating females with metoclopramide-induced HPRL and physiological HPRL after weaning to monitor the evolution of EGP and bone tissue.

Although the interpretation of the present data has some limitations with regard to its correlation with human biology, these findings suggest that hyperprolactinemia can have multiple effects on bone metabolism, and understanding their relationship is expected to be very helpful in the management of patients with hyperprolactinemia aside from infertility. However, few studies have investigated the prevalence of osteoporosis and fractures in patients with hyperprolactinemia**,** which is why animal models are greatly important. For this reason, future studies must be carried out to deepen this research by using electron microscopy and molecular biology tools to provide answers to the various questions that have arisen.

## Conclusion

The present study revealed excess cell death (TUNEL) of chondrocytes, consisting of 2-fold in the EGP of the femur and 4-fold in the EGP of the tibiae of a lactating female with hyperprolactinemia induced by metoclopramide, with greater involvement of the tibiae in relation to the femur, which is a fact that can lead to an imbalance between chondrogenesis and osteogenesis.

## Ethics approval and consent to participate

In accordance with the Brazilian College of Animal Experimentation. Approved by the Committee of Ethics on Animal Experimentation Institutional of UNIFESP and the Review Board (Report n° 6082130319).

## Authors’ contributions

Design of the study (ASLA, TAR, RCTG, MJS); conduct of the study (ASLA, RCTG); interpretation of the data (ASLA, RCTG, JMSJr, OPAJr); preparation and review (ASLA, RCTG) or approval of the manuscript (ASLA, RCTG, MJS).

## Funding

Nothing to declare.

## Conflicts of interest

The authors declare no conflicts of interest.
